# Relationships Between Distention-, Butyrate- and Pellet-Induced Stimulation of Peristalsis in the Mouse Colon

**DOI:** 10.3389/fphys.2020.00109

**Published:** 2020-02-18

**Authors:** Wei Tan, Grace Lee, Ji-Hong Chen, Jan D. Huizinga

**Affiliations:** ^1^Department of Gastroenterology, Renmin Hospital of Wuhan University, Wuhan, China; ^2^Department of Medicine, Division of Gastroenterology, Farncombe Family Digestive Health Research Institute, McMaster University, Hamilton, ON, Canada

**Keywords:** butyrate, propionate, peristalsis, peristaltic reflex, colonic motility

## Abstract

**Background/Aims:**

Luminal factors such as short-chain fatty acids are increasingly recognized for playing a regulatory role in peristaltic activity. Our objective was to understand the roles of butyrate and propionate in regulating peristaltic activity in relation to distention-induced activities.

**Methods:**

Butyrate and propionate were perfused intraluminally under varying intraluminal pressures in murine colons bathed in Krebs solution. We used video recording and spatiotemporal maps to examine peristalsis induced by the intrinsic rhythmic colonic motor complex (CMC) as well as pellet-induced peristaltic reflex movements.

**Results:**

The CMC showed several configurations at different levels of excitation, culminating in long distance contractions (LDCs) which possess a triangular shape in murine colon spatiotemporal maps. Butyrate increased the frequency of CMCs but was a much weaker stimulus than distention and only contributed to significant changes under low distention. Propionate inhibited CMCs by decreasing either their amplitudes or frequencies, but only in low distention conditions. Butyrate did not consistently counteract propionate-induced inhibition likely due to the multiple and distinct mechanisms of action for these signaling molecules in the lumen. Pellet movement occurred through ongoing CMCs as well as pellet induced peristaltic reflex movements and butyrate augmented both types of peristaltic motor patterns to decrease the amount of time required to expel each pellet.

**Conclusions:**

Butyrate is effective in promoting peristalsis, but only when the level of colonic activity is low such as under conditions of low intraluminal pressure. This suggests that it may play a significant role in patients with poor fiber intake, where there is low mechanical stimulation in the lumen.

## Introduction

Colonic motility is dependent on stimuli, and colonic distention is a major stimulus for the initiation of motor patterns. Recently, high-resolution spatio-temporal mapping has given us more insight into motor patterns in the mouse colon (Roberts et al., 2007, 2008; Vincent et al., 2018; Beck et al., 2019) the rabbit colon (Dinning et al., 2014; Chen et al., 2016) and the human colon (Dinning, 2016; Chen et al., 2017, 2018; Corsetti et al., 2017). Despite the fact that luminal stimulation is critical for normal colonic motility, the role of luminal factors in controling or modifying motility has not been extensively studied. When digested, content leaves the small intestine and enters the colon. The content causes distension of the colon and/or increases in luminal pressure that evokes motor activity; at the same time, microbial fermentation produces metabolites that might activate [e.g., butyrate (Hurst et al., 2014), 5-HT (Fukumoto et al., 2003)] or inhibit [e.g., propionate (Hurst et al., 2014)] motility. Hence under normal conditions, the colon appears to find a balance between different stimuli to generate motor patterns that facilitate digestion, absorption, and microbiota homeostasis. Consequently, poor colonic motility may be caused by an imbalance in these factors. In support of this hypothesis, we recently found that abnormal motor patterns in germ-free mice can be reversed to normal by luminal butyrate (Vincent et al., 2018). Our goal for the present study was to understand how butyrate, a natural short chain fatty acid (SCFA) in the colon, modulates peristaltic activity by evaluating its effects in relation to both distention and propionate, another natural SCFA that appears to have effects opposite to that of butyrate. Knowledge of butyrate’s effects could indicate whether its potential roles can be leveraged as an intraluminal prokinetic in motility disorders.

In order to fully understand the effect of SCFAs on the generation of peristaltic motor patterns, we have to distinguish two types of peristalsis. Peristalsis can occur through the generation of a neurally induced rhythmic motor pattern, the colonic motor complex (CMC) (Corsetti et al., 2019), previously referred to in the literature as the colonic migrating motor complex in the mouse colon (Fida et al., 1997; Bayguinov et al., 2010) or the peristaltic wave (Smith et al., 2003) or cyclic motor complex in the guinea pig colon (Costa et al., 2017), long distance contractions (LDCs) in the rat (Chen et al., 2013) and rabbit (Chen et al., 2016) or mass peristalsis in the rabbit colon (Lentle et al., 2008). The CMC can be evoked in the empty colon by an increase in intraluminal pressure in part or all of the colon. The CMC is a neurogenic motor pattern, yet the myogenic control system is an integral part of this complex (Bayguinov et al., 2010; Huizinga et al., 2011; Chen et al., 2013; Costa et al., 2013; Smith et al., 2014). The CMC involves the activation of interstitial cells of Cajal (Huizinga et al., 2011; Smith et al., 2014); Bayguinov et al. (2010) showed that it is dependent on a complex integration between myenteric neuronal and ICC-MP networks. The CMC is not dependent on slow wave activity from ICC-DMP (Yoneda et al., 2002, 2004; Spencer et al., 2019), but it involves stimulus dependent pacemaker activity from ICC-MP (Bayguinov et al., 2010).

Peristalsis can also be evoked by a local luminal stimulus such as a pellet in the colon of a mouse or rabbit or aggregated contents in the human colon (Spencer et al., 2016). This is a reflex that can be called the Bayliss and Starling reflex (Bayliss and Starling, 1900; Spencer et al., 1999), and it was suggested that this reflex occurs because of the polarity of enteric neurons (Bornstein et al., 2004): pellets activate cholinergic excitatory neurons to stimulate contractions oral to the pellet, while simultaneously activating inhibitory nitrergic neurons anal to the pellet (Spencer et al., 2002; Dickson et al., 2008). Nevertheless, it is not a simple reflex; bolus size and consistency affect propulsion speed, indicating that the features of the bolus-induced peristatic movement adapt to conditions (Costa et al., 2015). Although the neural component of the reflex is obvious, a role for a myogenic component, in particular related to the oral to anal direction of transit is likely (Huizinga et al., 2011; Sia et al., 2013). It was our objective to understand a potential role for butyrate in both of these mechanisms that can initiate peristalsis.

## Materials and Methods

### Animals

All animal procedures were approved by the McMaster University Animal Research Ethics Board (AUP:14-12-49). 8–10-week-old female C57BL/6 mice were purchased from Jackson Laboratories. The animals were fed *ad libitum* and housed under standard conditions (22 ± 2°C; 12/12-hr light/dark cycle) for at least 1 week prior to experiments.

### Colon Preparation

C57BL/6 mice were euthanized by isofluorane inhalation followed by cervical dislocation. After opening the abdominal cavity, the whole colon was carefully excised and placed in oxygenated (95% O_2_ and 5% CO_2_) Krebs solution at 4°C. The mesentery, blood vessels, and cecum were removed by ophthalmic scissors, and the fecal contents were flushed out by gavage with Krebs solution. Care was taken throughout these steps to minimize any stretching and disruptions to the mucosal layer.

#### Colonic Motility and Pressure Recording

**FIGURE 1:**
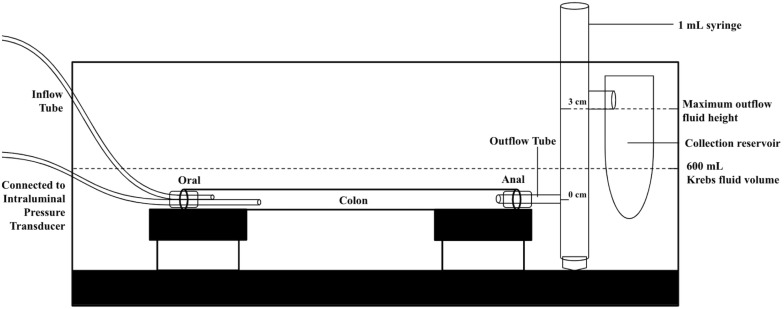
Schematic of the colon bath setup to maintain 3 cm outflow pressure. For another experimental condition, the 1 mL syringe was replaced by an open collection reservoir to create 0 cm of outflow pressure.

The whole colon was transferred into the organ bath, which was filled with 600 ml of oxygenated (95% O_2_ and 5% CO_2_) Krebs solution. The temperature of this Krebs solution was kept at 35°C through a heating tube which circulated water from an external water heater. The oral and anal ends were cannulated with an 18G needle and then fixed to the platform attached to the floor of the organ bath (Figure 1). The setup was also similar to a previously published photo (Parsons and Huizinga, 2015) and schematic drawing (Beck et al., 2019), for further reference. The oral end was connected to two plastic tubes, and the anal end was attached to a 1 ml syringe without the plunger. At the anal end, each CMC caused outflow and was followed by minimal back flow. The pressure from fluid accunulating in the 1 mL syringe and its back flow was used to simulate endogenous conditions of increasing intraluminal pressure. The outflow height was allowed to reach a maximum of 3 cm above the anal end before any additional fluid was drained into a collection reservoir. At that point, fluid levels in the syringe fluctuated between 2.8 and 3 cm at most. At the oral end, one tube continuously perfused the colon with Krebs solution, while the other was connected to an intraluminal pressure transducer (Figure 1). This pressure port was located at the proximal-middle colonic junction (about 1/3 from oral end) and the transducer was connected to a Grass LP 122 amplifier (Astro-Med, Brossard, QC, Canada). Its pressure signal was digitized using a MiniDigi 1A A-D converter, which was then displayed via Axoscope 10 software (Pclamp 10 software, Molecular Devices, Toronto, ON, Canada). The inflow perfusion was pumped into the lumen by a peristaltic pump (P-1, Pharmacia, Sweden) at an inflow rate of 30 μl/min.

A miniature CDD camera (Effio CCD 700TVL, SONY, Hong Kong, China) was fixed above the preparation and used to record diameter changes indicative of colonic motility. The resulting videos were transferred to ImageJ for further analysis through a DMapLE plugin written by Dr. Sean Parsons. This software generated spatiotemporal maps of diameter changes (due to circular muscle contraction) over time. The diameter was assessed by measuring the distance between the two contrast borders at both sides of the colon (contrast between a white colon and black background). The colonic width was calculated at each point along the its length (image *Y*-axis), for each video frame (image *X*-axis) to generate the final DMap image. We used a gray scale with white as relaxation or distention and black as contraction (reduction in colon diameter).

### Intraluminal Solutions

Krebs solutions contained (in mM): 120 NaCl, 6 KCl, 15.5 NaHCO_3_, 1.2 NaH_2_PO4, 0.1 citric acid, 0.1 aspartic acid,153 2.5 CaCl_2_, 1.2 MgCl_2_, and 6 glucose. A phosphate buffer solution (PBS) was used as an intraluminal inflow fluid, which contained (in mM): 117 NaCl, 3.9 KCl, and 10 Na_2_HPO_4_. Since glucose and high inflow rates can also induce motor activity through releasing 5-HT, PBS and low inflow rates provided the optimal conditions for viewing the effects of butyrate. 1 mM, 10 mM, 30 mM, and 100 mM of sodium butyrate were dissolved into PBS solution, and Tris buffer was used to adjust pH values (7.35–7.45). Addition of 1–100 mM NaCl to PBS solution, did not change motor activity, consistent with a study by Hurst et al. (2014). Kendig et al. (2014) also did not observe an effect of osmolarity on pellet propulsion in the rat.

### Experimental Design: General Distention and Butyrate Experiments

After a 30-min acclimation period, colonic motor activity and intraluminal pressure changes were recorded. Two forms of general distention were used, by allowing the outflow tube to drain into an empty 1 ml syringe placed vertically, its bottom flush with the colon. First, “increasing outflow pressure” was created by allowing all expelled Krebs solution to accumulate in the 1 mL outflow syringe. This fluid’s rising height exerted increasing intraluminal pressure and distention over time. Second, we created “fixed outflow pressures” by maintaining the fluid height at 0 cm or 3 cm above the level of the colon.

For baseline, the outflow pressure was fixed at 0 cmH_2_O and activity recorded for 15 to 20 min. Interventions consisted of various solutions of butyrate and/or propionate added under both fixed and increasing outflow pressures. Activity under fixed outflow pressure was recorded for 15 to 20 min, and activity for increasing outflow pressure was recorded for 40 min to allow for maximal fluid accumulation. The colon was given 10 min to acclimatize after each alteration in butyrate or propionate solutions.

### Experimental Design: Pellet-Induced Distention Experiments

Artificial pellets of varying sizes (length: 10 mm, diameter: 1.5–3 mm) were made by silicone putty (EasyMold, Environmental Technology Inc., Fields County, CA, United States) and coated with black nail polish to ensure their visibility on the spatiotemporal maps. For each colon, the artificial pellet was size-matched with the endogenous pellets that were extracted during the cleaning process. Unlike with previous experiments, the colon was pinned directly to the floor of the organ bath, with its oral and anal ends kept open. After a 30-min acclimatization period, the baseline activity of the colon was recorded for 15 min. A pellet was inserted into the oral end and its movements through the colon were recorded for 15 min or until it was expelled, upon which additional pellets would be inserted for a maximum of 30 min. In other experiments, a polyethylene tube was inserted into the distal colon to allow perfusion with Krebs solution with or without butyrate for 15 min. The pellet was then re-inserted under the aforementioned conditions and its movements recorded.

#### Data Analysis and Statistical Methods

All averaged values are presented as mean ± SEM for parametric distributions, and median with interquartile range (Q25, Q75) for non-parametric distributions. Statistical tests for significance were performed with SPSS (Chicago, Ill. United States) and Real Statistics (Charles Zaiontz). In Tables 1, 2, and 4, one-way ANOVA with Tukey’s *post hoc* tests were used to derive specific *P* values. For Tables 3 and 5, the differences in expulsion time and the number of pellet movements were compared using Student’s Independent *T* tests, with equal variances. The differences between the frequencies of CMCs and frequencies of reflexes in the pellet experiments (Tables 3 and 5) were compared by Wilcoxon–Mann–Whitney tests, using exact *P* values. To compare the effects of butyrate at 0 cm vs. 3 cm of fixed distention, Student’s Independent *T* tests with equal variances were used. In the sudden distention experiments, the Student’s Paired *T* test was used to compare the same sample before and after an intervention.

**TABLE 1:** The effect of increasing distention induced by outflow pressure on CMC frequency and diameter of the colon.

	0 min	10 min	20 min	30 min	40 min
Frequency of CMCs (cpm) (*N* = 7)	0	0.19 ± 0.07	0.33 ± 0.07	0.49 ± 0.03	0.44 ± 0.04^a^
Diameter (mm) (*N* = 7)	1.66 ± 0.06	1.73 ± 0.05	1.92 ± 0.06	2.05 ± 0.08	2.21 ± 0.11^b^
CMC pressure amplitude (cmH_2_O) (*N* = 7)	0	3.70 ± 0.75	5.94 ± 1.19	7.82 ± 1.52	8.33 ± 1.14^c^
Outflow pressure (cmH_2_O) (*N* = 7)	0	1.44 ± 0.13	3.46 ± 0.20	5.37 ± 0.31	6.86 ± 0.24^d^

*^*a*^*P* = 0.017, ^*c*^*P* = 0.048, ^*d*^*P* < 0.001 compared to the group at 10 min. ^*b*^*P* < 0.001 compared to the group at 0 min. Data are presented as means ± SEM. *P* values were determined by a one-way ANOVA and Tukey’s post hoc test.*

**TABLE 2:** Velocities of motor patterns with and without Krebs perfusion.

Group	Number of pellets inserted	Pellet expulsion (number)	Velocity of pellet movements (mm/s)	Velocity of CMCs (mm/s)	Velocity of reflex movements (mm/s)
No Krebs (*N* = 7)	20	Yes (13)	0.32 ± 0.03^a^	0.34 ± 0.03	0.20 ± 0.04
			(*n* = 56)	(*n* = 47)	(*n* = 9)
		No (7)	0.18 ± 0.01	0.21 ± 0.01	0.06 ± 0.01
			(*n* = 37)	(*n* = 30)	(*n* = 7)
Krebs	41	Yes (27)	0.39 ± 0.03	0.41 ± 0.03	0.37 ± 0.04
(*N* = 17)			(*n* = 76)	(*n* = 26)	(*n* = 50)
		No (14)	0.29 ± 0.02	0.35 ± 0.03	0.23 ± 0.02
			(*n* = 34)	(*n* = 16)	(*n* = 18)

*^*a*^*P* = 0.012 for the velocity of expelled vs. non-expelled pellet movements without Krebs perfusion. Velocities are presented as mean ± SEM. *P* values were determined by a one-way ANOVA and Tukey’s post hoc test.*

**TABLE 3:** Frequencies of motor patterns with and without Krebs perfusion.

Group	Number of pellets	Expulsion Time (min)	Number of pellet movements	Frequency of CMCs (cpm)	Frequency of reflex movements (cpm)
No Krebs (*N* = 7)	13	15.4 ± 2.2 (*n* = 13)	4.3 ± 0.5 (*n* = 13)	0.21 (0.18, 0.29) (*n* = 13)	0.06 (0.05, 0.11) (*n* = 7)
Krebs (*N* = 17)	27	7.5 ± 0.9^a^ (*n* = 27)	2.8 ± 0.2^b^ (*n* = 27)	0.16 (0.12, 0.23) (*n* = 14)	0.25 (0.14, 0.68)^c^ (*n* = 23)

*^*a*^*P <* 0.001, ^*b*^*P* = 0.002, ^*c*^*P* = 0.001 compared to No Krebs. Values are presented as mean ± SEM for parametric distributions or median (Q25, Q75) for non-normal distributions. *P* values for Time and Number of Pellets were determined by Student’s independent *T* tests with equal variances. *P* values for Frequencies of CMC and Reflex Movements were determined by Wilcoxon–Mann–Whitney tests.*

**TABLE 4:** Velocities of motor patterns in the control and butyrate groups.

Group	Number of pellets inserted	Pellet expulsion (number)	Velocity of pellet movements (mm/s)	Velocity of CMCs (mm/s)	Velocity of reflex movements (mm/s)
Control (*N* = 10)	21	Yes (16)	0.34 ± 0.03 (*n* = 49)	0.41 ± 0.38 (*n* = 17)	0.30 ± 0.04 (*n* = 32)
		No (5)	0.28 ± 0.04 (*n* = 9)	0.45 ± 0.05 (*n* = 2)	0.23 ± 0.05 (*n* = 7)
Butyrate (*N* = 10)	23	Yes (21)	0.39 ± 0.03 (*n* = 56)	0.42 ± 0.05 (*n* = 20)	0.38 ± 0.04 (*n* = 36)
		No (2)	0.34 ± 0.02 (*n* = 3)	–	0.34 ± 0.02 (*n* = 3)

**P* values (NS) were determined by a one-way ANOVA and Tukey’s *post hoc* test. Values are presented as mean ± SEM.*

**TABLE 5:** Frequencies of motor patterns in the control and butyrate groups.

Group	Number of pellets inserted	Expulsion Time (min)	Number of pellet movements	Frequency of CMCs (cpm)	Frequency of reflex movements (cpm)
Control (*N* = 10)	16	8.4 ± 1.0 (*n* = 16)	3.1 ± 0.3 (*n* = 16)	0.14 (0.12, 0.19) (*n* = 10)	0.19 (0.12, 0.40) (*n* = 15)
Butyrate (*N* = 10)	21	5.6 ± 0.9^a^ (*n* = 21)	2.7 ± 0.3 (*n* = 21)	0.32 (0.24, 0.43) (*n* = 4)	0.46 (0.30, 0.66)^b^ (*n* = 18)

*^*a*^*P* = 0.043 compared to Control. ^*b*^*P* = 0.036 compared to Control. Values are presented as mean ± SEM for parametric distributions or median (Q25, Q75) for non-normal distributions. *P* values for Time and Number of Pellets were determined by Student’s independent *T* tests with equal variances. *P* values for Frequencies of CMC and Reflex Movements were determined.*

One-way ANOVA with Tukey’s *post hoc* tests were used for all *P* value calculations involving propionate, except for the frequency of CMCs at the baseline and after 30 mM of propionate, where *P* values were calculated by Student’s Independent *T* tests assuming equal variances. A *P* value of 0.05 is considered to indicate significance. Figures were created with GraphPad PRISM software (La Jolla, CA, United States).

### Calculations

The diameter of the colon at any time was taken from the *z* axis of its spatiotemporal map. CMCs and reflexes were analyzed in terms of their amplitudes, frequencies and velocities. The amplitudes of CMCs and reflexes were calculated by measuring the maximum change in diameter at a fixed point along the length of the colon during each motor pattern. CMC frequencies and reflexes were calculated by dividing the number of motor patterns by the minutes over which they occurred. The velocities were calculated by taking the slope of a diagonal line drawn from the start to end of a spatiotemporal map of the motor pattern, located on a *y* axis indicating distance and an *x* axis indicating time.

Additionally, measures of pressure were also taken, including the outflow pressure, basic intraluminal pressure and pressure amplitude. The outflow pressure was recorded by examining the fluid level in the outflow syringe which had accumulated above the level of the colon (0 cmH_2_O). The basic intraluminal pressure was the lowest intraluminal pressure reading in each recorded session. Intraluminal pressure amplitude was calculated as the difference between the maximum and minimum intraluminal pressure values during any given motor pattern.

### Nomenclature

The nomenclature used for colonic propulsive motor patterns in animal models is not consistent in the literature. A major propulsive motor pattern is a pan-colonic rhythmic activity that has been called, amongst others, “colonic migrating motor complex,” “mass peristalsis” or “giant contractions” occurring at a frequency around 0.5 cpm in the mouse (Powell et al., 2003) and rabbit colon (Quan et al., 2017) and up to 2 cpm in the guinea pig (Costa et al., 2015). At a consensus meeting it was agreed to use the term “CMC” (Corsetti et al., 2019). We recently demonstrated in the rabbit colon that the characteristics of this motor complex are dramatically different at different levels of excitation (Shokrollahi et al., 2019). In the rabbit, mouse and rat, we and others have identified the long distance contraction (LDC) as the most forceful circumferential propulsive contraction evoked by distention that propels content down the colon; it is the most forceful expression of the CMC (Chen et al., 2013; Kendig and Grider, 2015; Yu et al., 2015; Li et al., 2016; Vincent et al., 2018). The propulsive nature of the LDC can be measured by its association with 3–5 ml outflow (Chen et al., 2016). LDCs have a characteristic triangular shape in spatiotemporal maps, very similar in the rabbit, mouse and rat, due to the fact that the most proximal part of the colon remains contracted while the front of the contraction propagates anally, preceded by relaxation (Chen et al., 2013). LDCs are neurogenic in that they are abolished by TTX or hexamethonium (Lentle et al., 2008; Chen et al., 2016). The second most prominent propulsive activity, observed under less excited conditions is the fast propagating contraction (FPC), often occurring in clusters. FPCs are circular muscle contractions that occur at a higher frequency and propagate at a higher velocity compared to LDCs and propagate usually in antegrade direction. They are less forceful than LDCs and they may occur in clusters of several FPCs (Quan et al., 2017). FPCs are also called “fast phasic activity” (Lentle et al., 2008) and clusters of FPCs have been associated with 1–3 ml outflow per FPC (Chen et al., 2016). In the rabbit colon it has been determined that single FPCs are myogenic (Lentle et al., 2008), but the forming of FPC clusters is neurogenic (Chen et al., 2016). LDCs and FPCs are two manifestations of CMCs that will be described in this paper.

In addition to these two motor patterns, there are three other motor patterns in the mouse colon, previously characterized in the rat colon: myogenic ripples (Chen et al., 2013), proximal rhythmic contractions (Li et al., 2016) and distal rhythmic propagating motor complexes (Chen et al., 2013; Li et al., 2016).

## Results

### The Motor Patterns of the Mouse Colon at Different Levels of Excitation

**FIGURE 2:**
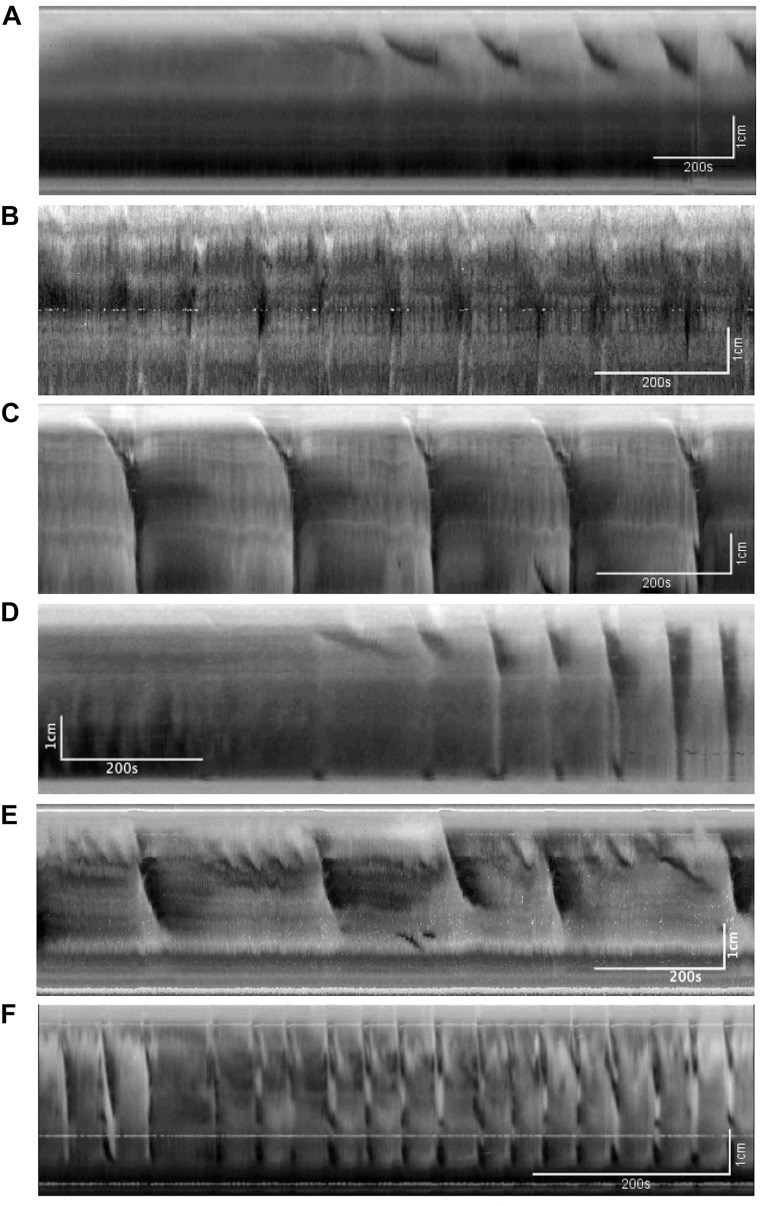
The colonic motor complex (CMC) at different levels of excitation. In this and all subsequent figures, black identifies contraction, and white relaxation or distention; the top of all figures is the proximal part of the colon. **(A)** Upon mild excitation by increased intraluminal pressure, proximal rhythmic (pacemaker) activity appear in the colon always starting in the most proximal pacemaker activity. These are usually rhythmic, and almost always start in the proximal colon to propagate a short distance. **(B)** Fast propagating contractions (FPCs). Further excitation may elicit a CMC, which is always rhythmic and triggered by proximal activity. The weakest form of CMCs presented as single FPCs, clustered-FPCs or a mix of short contractions that together form a complex of pan-colonic activity. **(C)** Long distance contractions (LDCs). Full-blown LDCs are shown here, which are manifestations of CMCs at their highest level of excitation. **(D)** This figure shows how proximal rhythmic contractions can develop from proximal activity to eventually trigger LDCs. **(E)** This figure shows how proximal activity can occur rhythmically and at a high frequency where only every 3rd or 5th activity triggers an LDC. **(F)** “Broken” long distance contractions. The CMC can show many compositions in between low-amplitude FPCs and full-blown LDCs. Here we see many LDCs we have previously identified as “broken LDCs” (Chen et al., 2013), where it appears that the LDC is interrupted by one or many streaks of inhibition.

Figure 2 shows examples of the CMC at different levels of excitation. These motor complexes are all pan-colonic and occur in a rhythmic manner. Without any stimulation, the mouse colon often exhibited rhythmic proximal activity (Figure 2A). Colons that were placed in the organ bath without any stretch or distention showed CMC activity in all preparations (Figure 2B). The weakest form of CMCs presented as single FPCs, clustered-FPCs or a mix of short contractions that together formed a complex of pan-colonic activity. Upon further distention, these weaker motor patterns then changed into various configurations with increasing distention, up to its most excited configuration, the LDC (Figures 2A–F).

### Distention-Induced Changes in the Colonic Motor Complex

Distention-mediated excitation was accomplished in three different ways.

#### The Colon Was Perfused With Krebs Solution and the Outflow Collected in a Vertical Tube so That the Intraluminal Pressure Gradually Increased

**FIGURE 3:**
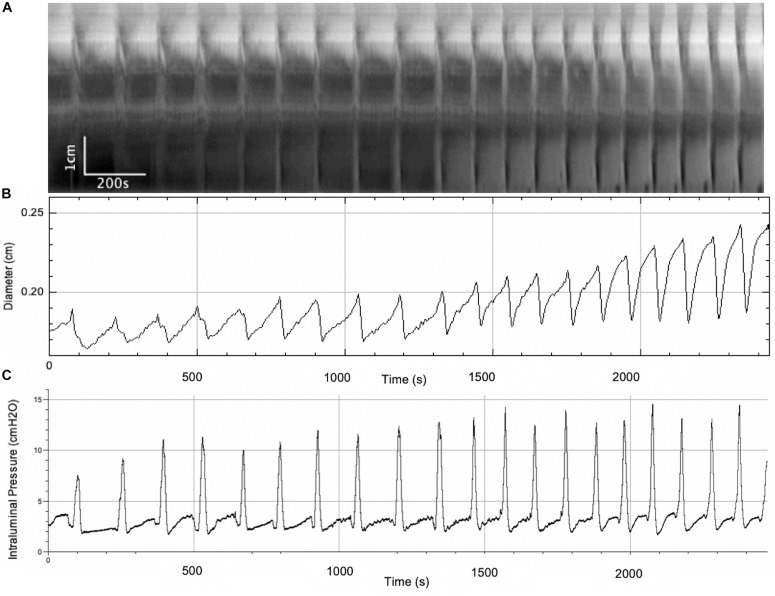
A gradual increase in intraluminal pressure causes an increase in CMC frequency and the amplitude of accompanying phasic contractions, as well as intraluminal pressure changes. **(A)** Spatiotemporal map of CMCs. **(B)** Average diameter of the colon over time. **(C)** Intraluminal pressure in the colon over time.

In this series of experiments, the outflow pressure increased gradually from 0 to ∼ 7 cmH_2_O (*N* = 7) (Figure 3 and Table 1). The resultant change in mean intraluminal pressure did not increase to the same degree: the mean intraluminal pressure started at 1.47 ± 0.43 and increased to 4.16 ± 0.59 cmH_2_O, likely due to adaptive relaxation in the colon (*N* = 7). With increasing intraluminal pressure, the CMCs progressed from weak simultaneous contractions to FPCs, culminating in LDCs (Figures 2C,D, 3). The CMC pressure amplitude increased from 3.70 ± 0.75 to 8.33 ± 1.14 cmH_2_O (*p* = 0.048, *N* = 7, *n* = 28), accompanied by a significant increase in the average frequency of CMCs from 0.19 ± 0.07 to 0.44 ± 0.04 cpm (*p* = 0.017, *N* = 7, *n* = 28) (Table 1).

#### The Colon Was Perfused With Krebs Solution With the Outflow Fixed at 0 cmH_2_O Above the Colon

**FIGURE 4:**
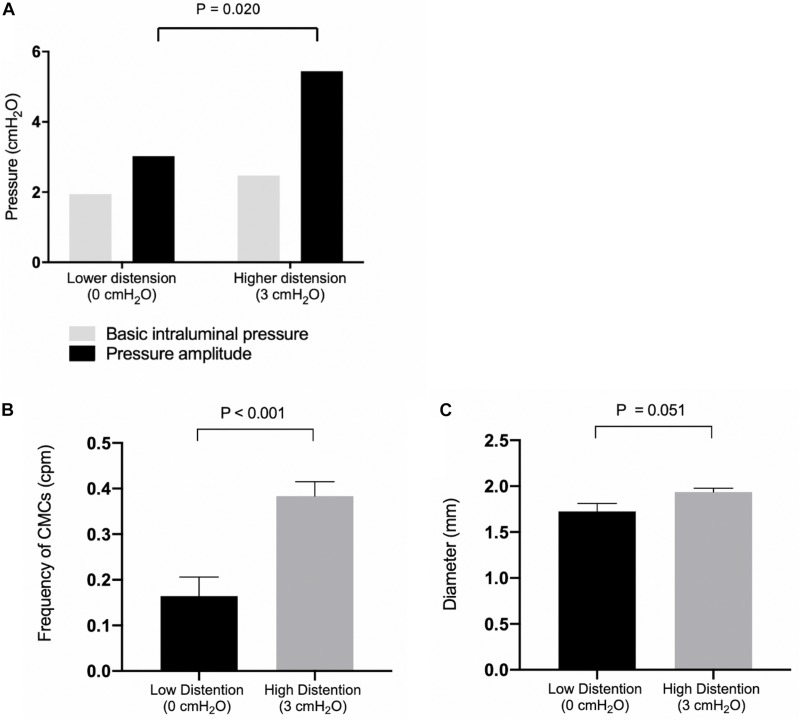
**(A)** Basic intraluminal pressure and pressure amplitudes of observed CMCs under low and high distention. Compared with 0 cmH_2_O (*N* = 7), 3 cmH_2_O (*N* = 5) of outflow pressure increased the pressure amplitude of CMCs observed (*P* = 0.020). **(B)** CMC frequencies under low and high distention. Compared with 0 cmH_2_O (*N* = 10), 3 cmH_2_O (*N* = 7) of outflow pressure increased the frequency of CMCs in the colon (*P* < 0.001. **(C)** Colonic diameter under low (*N* = 9) and high distention (*N* = 7). *P* values were determined by Student’s *T* test.

**FIGURE 5:**
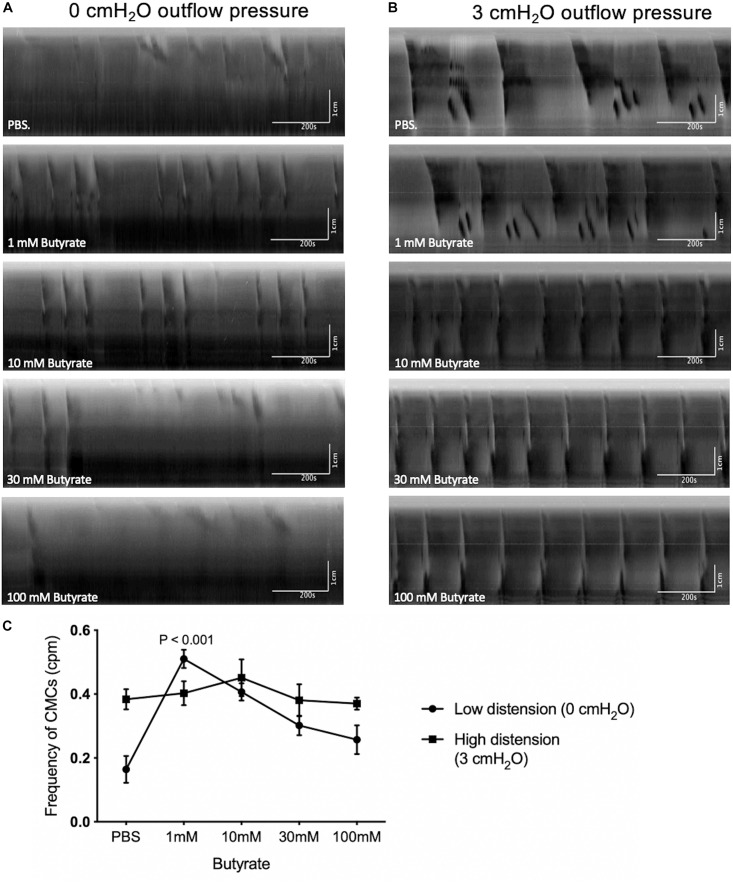
The effect of butyrate at fixed levels of high and low distention on CMCs. **(A)** Spatiotemporal maps showing the effects of increasing butyrate concentrations on colonic motor activity under low distention. Administering 1 mM of luminal butyrate caused proximal rhythmic contractions to extend into pan-colonic CMCs, specifically FPCs. LDCs were formed at 10 mM of butyrate but disappeared upon continued increases in butyrate concentration to 30 mM or 100 mM. **(B)** Spatiotemporal maps showing the effects of increasing butyrate concentrations on colonic motor activity under high distention. Administering 10 mM of butyrate increased CMC frequency on average. Further increases in CMC frequencies were inconsistent in response to 30–100 mM butyrate. **(C)** Frequency of CMCs in response to varying concentrations of butyrate in high and low distention. Butyrate (1 mM) significantly increases CMC frequency in instances of low intraluminal pressure and activity, as simulated by low distention (0 cmH_2_O of outflow pressure) (*N* = 10, *P* < 0.001). Increasing the concentration of butyrate to 10 mM, 30 mM or 100 mM did not further augment CMC frequency. Butyrate has a negligible effect on CMC frequency in instances of high intraluminal pressure and baseline motor activity, as simulated by high distention (3 cmH_2_O outflow pressure) (*N* = 7). *P* values were determined by a one-way ANOVA and Tukey’s *post hoc* test.

With outflow levels fixed at 0 cmH_2_O, the basic intraluminal pressure was 1.94 ± 0.44 cmH_2_O (*N* = 9, *n* = 7) due to a constant infusion of Krebs solution, and the average colonic diameter was 1.73 ± 0.09 mm (*N* = 9, *n* = 9) (Figure 4). Proximal rhythmic contractions and FPCs were the predominant motor patterns observed during this phase (Figure 5A), with LDCs present in 2 experiments.

Under these conditions, 1 mM butyrate increased the frequency of CMCs from 0.16 ± 0.04 to 0.51 ± 0.03 cpm (*p* < 0.001, *N* = 10, *n* = 42), but no significant increases in amplitude were observed (Figures 5A,C). 10 mM of butyrate also increased the frequency of CMCs from 0.16 ± 0.04 to 0.41 ± 0.03 cpm (*p* < 0.001, *N* = 10, *n* = 42) No significant changes in CMC frequency nor amplitude were found with additional butyrate by comparison (30 mM, 100 mM, Figure 5C).

#### The Colon Was Perfused With Krebs Solution With the Outflow Fixed at 3 cmH_2_O Above the Colon

When the outflow level was fixed at 3 cmH_2_O, the basic intraluminal pressure was 2.47 ± 0.90 cmH_2_O (*N* = 7, *n* = 5) with a diameter of 1.94 ± 0.04 mm (*N* = 7, *n* = 7, Figure 4). Under these conditions, all experiments exhibited pan-colonic motor complexes in the form of either FPCs or LDCs (Figure 5B). There was also a 2.42 ± 0.86 cmH_2_O increase (*p* = 0.019, *N* = 13, *n* = 13) in the pressure amplitude of CMCs and a 0.22 ± 0.06 cpm increase in the average frequency (*p* = 0.002, *N* = 17, *n* = 17) of pan-colonic CMCs in comparison to mice under 0 cmH_2_O of outflow pressure (Figures 4A,B).

Butyrate at 1–100 mM was administred to these preparation (*N* = 19) but none were capable of significantly increasing the frequency of CMCs observed (Figure 5B). Additionally, changes in CMC frequency and amplitude were not consistently correlated to increasing concentrations of butyrate during high distention (Figure 5C).

#### Transient Local Distention Occurring Due to a Sudden Blockage of Outflow

**FIGURE 6:**
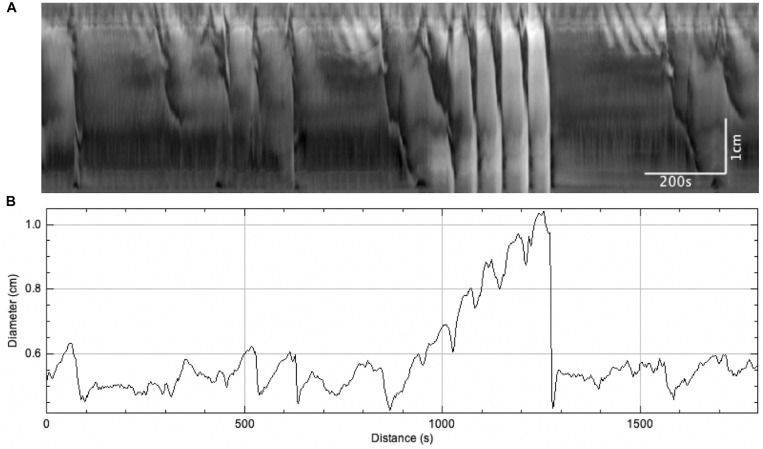
The effect of sudden distention on colonic motor activity caused by a blockage in outflow. When this occurred, LDCs immediately developed to expel the blockage and restore baseline activity levels. **(A)** Spatiotemporal map. **(B)** Average colonic diameter over time.

On three occasions, sudden local distention occurred in the middle of the colon due to a blockage of outflow by gas bubbles or dislodged mucosa (Figure 6A). The average diameter of the colon increased from 1.83 ± 0.29 to 2.73 ± 0.41 mm (*N* = 3, *n* = 6, *p* = 0.054) (Figure 6B). This local distention augmented colonic motor activity by generating (a) regular LDCs developing from proximal contractions or (b) strong LDCs from weak, pre-existing LDCs (Figure 6A). The frequencies of LDCs were significantly increased from 0.23 ± 0.04 to 0.60 ± 0.11 cpm (*N* = 3, *n* = 6, *p* = 0.035). When these blockages were spontaneously resolved, the previous weaker LDCs and proximal contractions returned.

### Butyrate-Induced Changes in the Colonic Motor Complex in the Presence of Propionate and Distention

#### Butyrate in the Presence of Propionate at 0 cmH_2_O of Outflow Pressure

**FIGURE 7:**
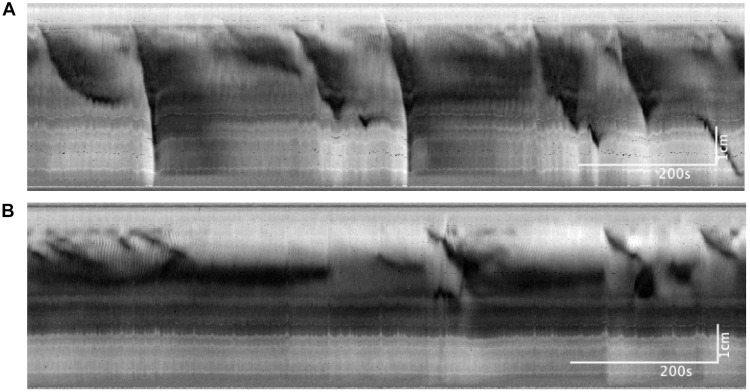
Colonic motor activity at baseline compared to 30 mM of luminally administered propionate. **(A)** Baseline. **(B)** Propionate (30 mM). Compared to baseline conditions, 30 mM propionate decreases LDCs and FPCs, while leaving proximal rhythmic activity intact.

In these experiments, the outflow pressure of the colon was maintained at 0 cmH_2_O, giving an average intraluminal pressure of 1.36 ± 0.20 cmH_2_O (*N* = 9). In this low-distention state, 30 mM of propionate decreased the average frequency of CMCs by 0.12 ± 0.05 cpm (*p* = 0.031, *N* = 9, *n* = 18). CMCs initially present were either completely abolished or reduced to proximal rhythmic contractions (Figure 7). Accordingly, their frequencies were either reduced to 0 cpm (*N* = 5), decreased by 0.13 ± 0.03 cpm (*N* = 2) or increased by 0.08 ± 0.06 cpm with lowered amplitudes (diameter reduction) by 1.55 ± 0.21 mm (*N* = 2).

**FIGURE 8:**
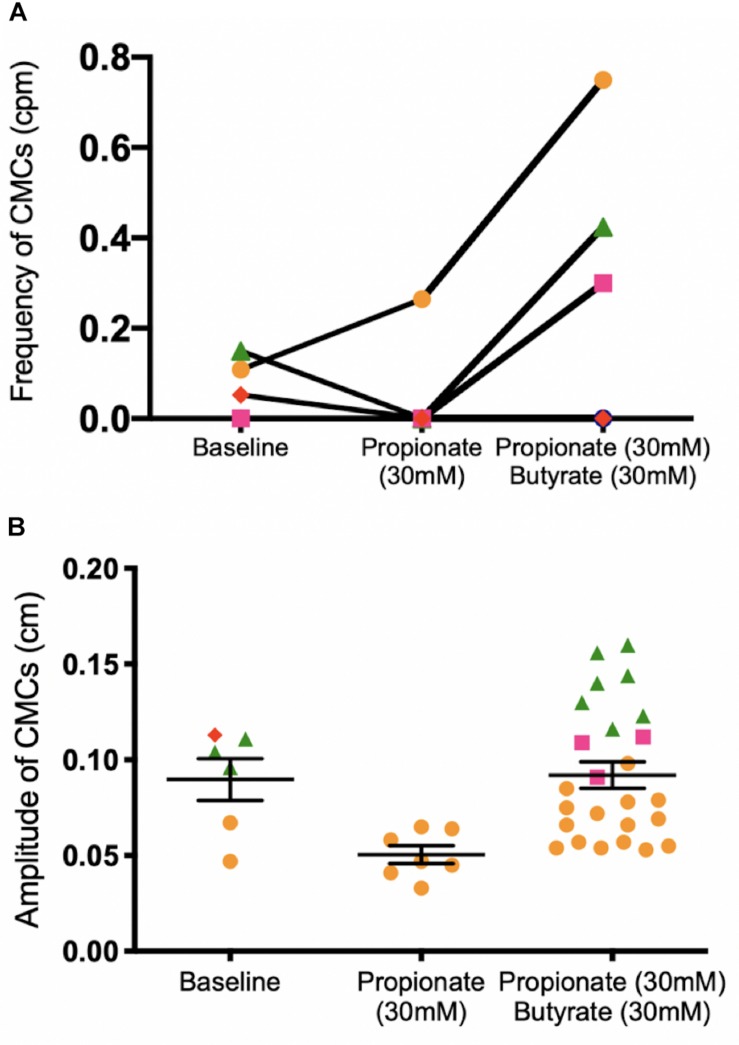
Colonic motor activity before and after adding 30 mM butyrate to a colon under low distention (0 cmH_2_O outflow pressure) and 30 mM propionate. **(A)** CMC frequency. **(B)** CMC amplitude. *N* = 5, *n* = 37 where N indicates number of mice (separated by color and shape), *n* indicates number of motor patterns (one data point). The ability for butyrate to increase the frequency of CMCs is inconsistent in the presence of propionate. In three mice (orange, green, pink), an increase in the number of observed CMCs corresponded to more data points after 30 mM butyrate was added to propionate. In one mouse, no CMCs were observed, corresponding to no data points in Figure 8B. *P* values (NS) were determined by a one-way ANOVA and Tukey’s *post hoc* test.

30 mM butyrate was added after 30 mM propionate perfused the colon in a low distention state, with an average intraluminal pressure of 2.79 ± 0.46 cmH_2_O induced by maintaining 0 cmH_2_O of outflow pressure (*N* = 5). Increases in CMC frequency of 0.40 ± 0.16 cpm were seen in 3 mice (*N* = 3). Overall, adding 30 mM butyrate to 30 mM propionate increased the amplitude of CMCs by 0.04 ± 0.01 cm (*p* = 0.009, *N* = 5, *n* = 32, Figure 8B). However, it had inconsistent effects on CMC frequency at a low distention state (Figure 8A).

#### Butyrate in the Presence of Propionate Under Increasing Distention Induced by Outflow Pressure

**FIGURE 9:**
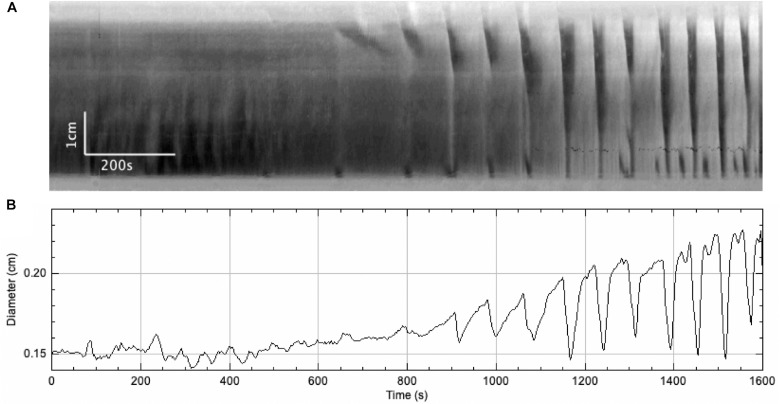
Colonic activity in response to increasing distention in the presence of 30 mM propionate. **(A)** Spatiotemporal map. **(B)** Average diameter over time. Distention is a strong stimulus which can easily overcome the inhibitory effects of propionate to generate strong LDCs.

**FIGURE 10:**
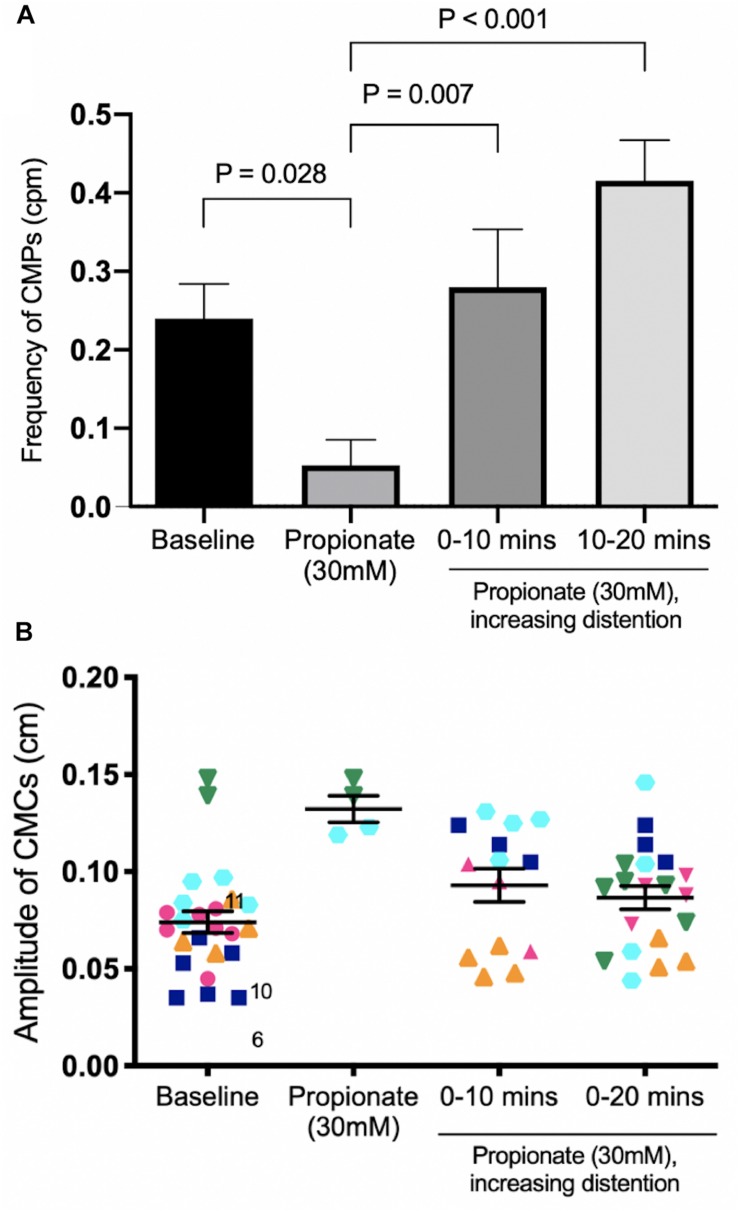
The effect of 30 mM propionate and increasing outflow pressure over 20 min. **(A)** CMC Frequency. Compared to 30 mM propionate alone, increasing outflow pressure with 30 mM propionate significantly increased the frequencies of CMCs at both 10 min and 20 min. **(B)** CMC Amplitude. *N* = 5, *n* = 62 where *N* indicates number of mice (distinguished by color and shape), *n* indicates the number of motor patterns (one data point). *P* values were determined by a one-way ANOVA and Tukey’s *post hoc* test.

In these experiments, the outflow pressure was allowed to accumulate from 0 to 3 or 5 cm. Motor patterns at baseline began as weak myogenic ripples or proximal rhythmic contractions. Increased distention induced strong, high amplitude proximal rhythmic contractions, which then progressed into weak CMCs in the form of FPCs, and finally LDCs at the highest level of distention (Figures 8, 9). CMC frequencies increased by 0.23 ± 0.07 cpm (*p* = 0.007, *N* = 6, *n* = 12) after 10 min and 0.36 ± 0.07 cpm after 20 min (*p* < 0.001, *N* = 6, *n* = 12) (Figure 10A). The amplitudes of CMCs increased by 0.058 ± 0.015 cm (*p* = 0.002, *N* = 6, *n* = 28) upon adding 30 mM propionate and decreased by 0.046 ± 0.015 cm (*p* = 0.020, *N* = 6, *n* = 24) after 20 min (Figure 10B).

**FIGURE 11:**
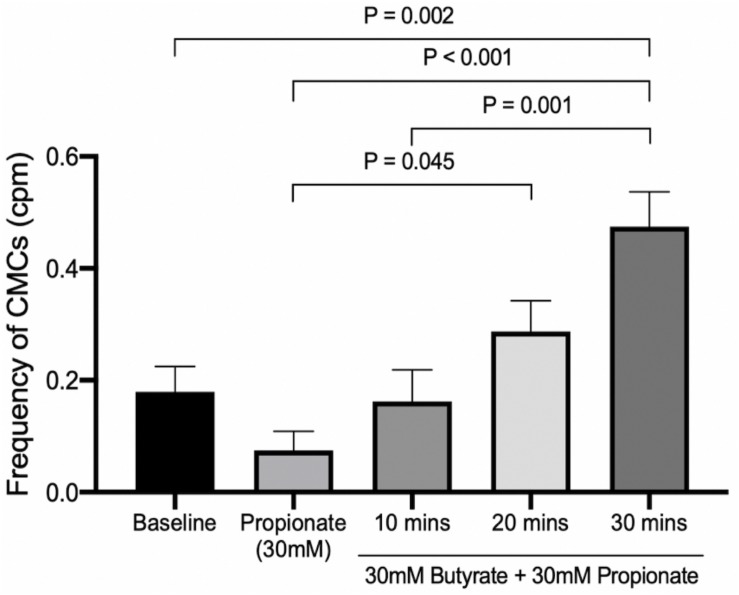
The effect of 30 mM butyrate on increasing mechanical distention’s ability to regenerate CMCs under 30 mM propionate. Since CMC frequencies increase in a pattern similar to that of Figure 10, the significant differences are more likely due to mechanical distention than the effects of butyrate. *P* values were determined by a one-way ANOVA and Tukey’s *post hoc* test.

30 mM butyrate was added with 30 mM propionate under increasing outflow pressure (0 to ∼3 or 5 cmH_2_O), as described above (*N* = 8). Compared to propionate alone, butyrate coupled with distention was able to significantly increase CMC frequencies 0.21 ± 0.07 cpm (*p* = 0.045, *N* = 8, *n* = 16) after 20 min (Figure 11). Butyrate surpassed baseline CMC frequencies by 0.30 ± 0.07 cpm (*p* = 0.002, *N* = 8, *n* = 16) after 30 min (Figure 11). However, these changes were not significantly different from those induced solely by distention in the presence of propionate.

### Motor Patterns Associated With Pellet Expulsion and Butyrate

**FIGURE 12:**
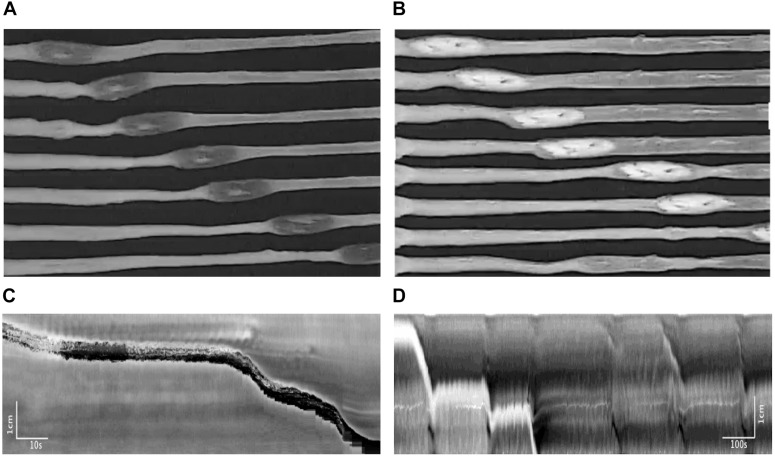
Motor patterns associated with pellet expulsion with Krebs perfusion. **(A)** Movement of the pellet into the distal colon by the peristaltic reflex. **(B)** Movement of the pellet into the distal colon by regularly occurring CMC. After traveling a partial length of the colon, the pellet may stop at one point for several minutes. It is eventually dislodged by intrinsic CMCs, which allow the pellet to become expelled at the anal end. Throughout this process, the frequencies of these CMCs do not change. **(C)** Spatiotemporal map of **(A)**. **(D)** Spatiotemporal map of **(B)**.

Pellet movements occurred through two distinctly different motor activities. Most pellet movements occurred through CMCs. Almost always, a CMC would move the pellet for a short distance and then the CMC would continue without the pellet. In addition, a second type of motor activity was induced by the pellet, termed the reflex movement, the classical Bayliss and Starling peristaltic reflex; a ring contraction developed orally to the pellet (Figure 12A), which was seen in the spatiotemporal maps as a black band (Figure 12B), which then moved the pellet (Bayliss and Starling, 1900). The pellet would stop moving once the ring contraction subsided. The ring contraction was often preceded by a relaxation, which can be observed in the spatiotemporal maps as a short retrograde movement of the pellet (Figure 12B). Rarely was the pellet moved out of the colon by a single CMC or a single reflex movement.

#### Pellet Movement in a Non-distended Colon Without Krebs Perfusion

**FIGURE 13:**
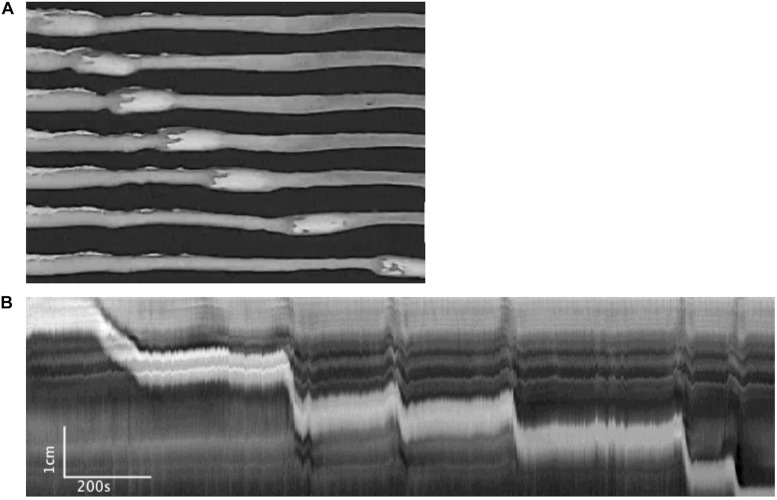
Motor patterns associated with pellet expulsion without Krebs perfusion. **(A)** Distention-mediated reflex movements propel the pellet partially through the colon, before intrinsic neurogenic CMCs eventually expel it at the anal end. **(B)** Spatiotemporal maps associated with **(A)**.

In seven mice, 20 pellets moved out of the colon within 15.40 ± 2.23 min, through 47 CMCs and nine reflex movements (Figure 13). When the pellet moved along the colon but did not expel within 30 min, the experiment was terminated; this occurred with seven pellets that moved through only 61 ± 2% of the length of the colon through 30 CMCs and seven peristaltic reflex movements. Specifics of these motor patterns are shown in Tables 2 and 3.

#### Motor Patterns Associated With Pellet Expulsion With Krebs Perfusion From the Distal End in the Absence of Butyrate

In 17 mice, 27 pellets moved out of the colon within 7.51 ± 0.92 min, through 26 CMCs and 50 reflex movements (Figures 12A–D and Tables 2, 3). The CMC frequency did not differ upon insertion of the pellets, but the frequency of reflex movements increased from 0.06 (0.04, 0.12) to 0.25 cpm (0.13, 0.74) (*p* = 0.001) (Table 3 and Figure 12D). CMCs which moved these pellets were also of higher amplitude compared to those either before or after pellet insertion (Figure 12D). Finally, introducing distention via Krebs perfusion significantly increased the velocity of the reflex movements (*p* = 0.012, Table 2).

#### Motor Patterns Associated With Pellet Expulsion With Butyrate

**FIGURE 14:**
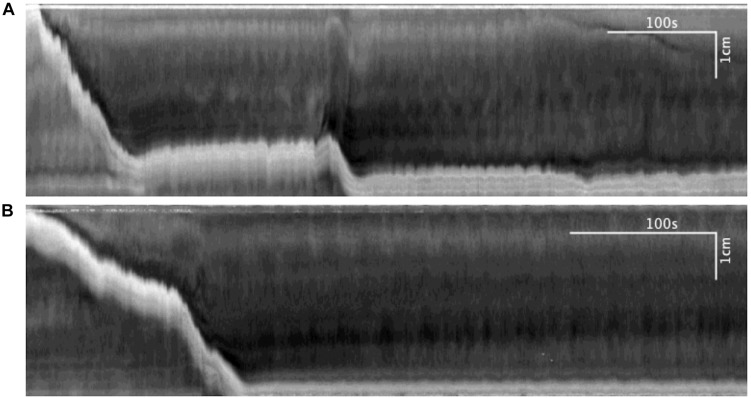
**(A)** Spatiotemporal map of control. **(B)** Spatiotemporal map of 30 mM butyrate on pellet propulsion.

The effect of butyrate on pellet expulsion was studied in colons with Krebs perfusion. Figure 14 shows changes in the peristaltic reflex movements in the presence of butyrate. When perfusion consisted solely of Krebs solution, 16 out of 21 pellets moved out of the colon in 30 min, compared to 21 out of 23 pellets with 10 mM of butyrate (Table 4). 3.06 ± 0.27 movements over 8.73 ± 1.00 min were required to expel each pellet with Krebs perfusion, whereas with butyrate it took 2.67 ± 0.28 movements over 5.62 ± 0.85 min (*p* = 0.043, Table 5). In the control group, 21 pellets moved out of the colon by 17 CMCs and 32 peristaltic reflexes, whereas with butyrate, 23 pellets moved out of the colon by 20 CMCs and 36 peristaltic reflexes (Table 4). The average velocity of peristaltic reflex movements did not differ between these two phases (0.30 ± 0.04 mm/s with Krebs and 0.38 ± 0.04 mm/s with 10 mM butyrate) (Table 4). Similar to the effect of Krebs perfusion, the reflex movements were augmented with an increase in frequency from 0.19 to 0.46 cpm by the introduction of butyrate (*p* = 0.036, *N* = 10, *n* = 23), while the frequency of CMCs remained similar.

## Discussion

Butyrate and propionate are primary SCFAs produced through the fermentation of indigestible carbohydrates, produced by specific subspecies of gut microbiota (Louis et al., 2016; Louis and Flint, 2017). These SCFAs are present in relative ratios of 1:1 in the human colon and have a potential role as signaling molecules that modulate colonic peristalsis (Hurst et al., 2014; Louis et al., 2016). Reigstad et al. (2015) demonstrated that enterochromaffin (EC) cells exposed to butyrate upregulate tryptophan hydroxylase-1 (Tph1), which catalyzes the formation of 5-HT, see also Martin et al. (2017). Although EC-released 5-HT is not essential for the generation of CMCs (Keating and Spencer, 2010; Vincent et al., 2018), experimental data do support a physiological role for this process (Smith et al., 2014). We sought to better understand the role of butyrate in regulation of colonic peristalsis and the present study found that although mechanical distention is by far the most powerful stimulus for CMC generation, butyrate has a modulatory excitatory role under all conditions studied.

### CMCs Are Intrinsically Present Under Minimal Mechanical Distention

Abundant literature exists describing colonic motor patterns in rabbits, guinea pigs, and rats, but those in the mouse remain relatively undetermined (Lentle et al., 2008; Chen et al., 2013; Costa et al., 2013; Shokrollahi et al., 2019). We first observed the mouse colon under baseline conditions to establish intrinsic motor patterns. Without stretch, distention or perfusion, proximal rhythmic contractions were often present, but were not classified as CMCs since they were not pan-colonic. In addition, low-amplitude CMCs in the form of clustered FPCs often occurred at a low cluster frequency, also shown in the rabbit colon (Chen et al., 2016). The presence of myogenic ripples indicates that ICC in the submuscular plexus (ICC-SMP) are also intrinsically active, with a hypothesized role in intestinal absorption (Huizinga et al., 2011).

Although CMCs are usually dependent on the presence of some intraluminal pressure, they can also occur in an empty colon without any intraluminal pressure as we show in the present study, albeit infrequently, under many experimental low distention conditions (Barnes et al., 2014). In this respect, the generation of a murine CMC is similar to that of human High-Amplitude Propagating Pressure Waves which occur without any apparent stimulus during High Resolution Colonic Motility when the colon is empty (Chen et al., 2017, 2018).

### Mechanical Distention Is a Strong Inducer of CMCs

As Shokrollahi et al. (2019) reported in rabbits, we observed that CMCs in the mouse colon progress in predictable phases depending on the level of excitation (Figure 2). Individual colons may begin with varying baseline activities along this spectrum, but mechanical distention will almost always increase CMC frequency, propagation distance and amplitude (Huizinga et al., 2011; Spencer et al., 2016). First, minimal distention enhances the amplitude and frequency of ICC-MP orchestrated FPCs, indicating that they are usually present but attenuated in the background (Figure 2A; Alberti et al., 2005). Upon further excitation, clusters of FPCs can merge to create high-amplitude LDCs, the highest level of CMC activity (Figures 2C,D).

A strong physiological stimulus in the human colon is general distention caused by digested food or feces. In the mouse, we mimicked this by using perfusion-generated gradual increases in intraluminal pressure. However, increases in intraluminal pressure do not correlate linearly with changes in distention, as our experiments show that adaptive relaxation minimizes increases in pressure as well as stretch. When adaptive relaxation is overcome, pressure and/or stretch become powerful stimuli for the generation of CMCs. This general stretch evokes rhythmic motor patterns which differ from those caused by localized stretch. Similar to the guinea pig ileum (Spencer et al., 1999), the “law of the intestine” is unlikely to be responsible for the rhythmic propulsive motor complexes in the colon (Huizinga et al., 2011). CMCs are generated instead by myenteric neural activity superimposed on ICC-MP generated slow waves (Pluja et al., 2001; Yoneda et al., 2004). In extreme cases it was found that CMCs can prevail without the submucosal plexus and mucosa, but not without the myenteric plexus and muscularis externa (Zagorodnyuk and Spencer, 2011). Nevertheless, under standard physiological conditions, luminal stretch involves both neuronal and muscular components by potentially activating mucosal sensory neurons to evoke activity in the myenteric plexus (Smith et al., 2014), although no direct electrophysiological recordings from sensory nerves in the mouse colon have been obtained yet.

### Butyrate Augments CMCs in Low Activity Conditions

Luminal butyrate at relatively low concentrations (1 mM) increased CMC frequencies when distention-induced activity was low, or when the outflow pressure was 0 cmH_2_O. When CMC activity was pronounced with higher levels of distention, such as at with 3 cmH_2_O outlflow pressure or when the outflow pressure was gradually increased, butyrate had no additional effects. Thus, it may be that butyrate does not have a significant role under normal physiological conditions, but its role might increase under conditions of low activity such as poor fiber intake where mechanical stimulation in the lumen is low. This butyrate action may occur by stimulating 5-HT production in enteroendocrine cells, which may act primarily on 5-HT_4_ receptors on sensory nerves (Hoffman et al., 2012; Yu et al., 2015; Reigstad et al., 2016).

### Butyrate Has Variable Effects on CMC Activity in the Presence of Propionate

Previous studies have postulated that propionate has an inhibitory effect on both short and full propagating contractions (Tazoe et al., 2008). In the present study, propionate was a weak inhibitor and any inhibition could be easily overcome by minor distention (Figure 9). Interestingly, the average frequencies of CMCs sometimes remained constant or increased after the administration of propionate, resulting in low-amplitude FPCs of moderate frequency. This corresponds to findings by Tazoe et al. (2008) who reported that 1–5 mM propionate could significantly increase the frequencies of “giant contractions” while decreasing their amplitudes by up to 400%.

Throughout our experiments, the effect of butyrate was characterized by an inconsistent capacity to overcome propionate’s inhibitory effects. Propionate and butyrate are endogenous ligands for two G-coupled protein receptors, FFAR2/GPR43 and FFAR3/GPR41. These receptors have been detected on enteroendocrine L cells throughout the GI tract, but a majority reside within the distal ileum and large intestine (Samuel et al., 2008). GPR41 couples with G_*i/o*_, is equally bound by propionate and butyrate, and has been shown to release peptide YY (PYY), thus playing a key role in lipolysis (Kaemmerer et al., 2010; Hurst et al., 2014). On the other hand, GPR43 is coupled to both G_*q*_ and G_*i/o*_ proteins and is preferentially bound by propionate over butyrate (Hurst et al., 2014). In addition to releasing PYY, this receptor is also involved in the downstream activation of mucosal mast cells and has been hypothesized to be involved in motility disorders associated with 5-HT signaling (Kaemmerer et al., 2010). The inhibitory effects of luminally administered propionate are accounted for by its activation of GPR41 and GPR43 to release PYY, an established satiety hormone that inhibits peristalsis and transit time. However, our experiments found that the capacity for butyrate to restore peristaltic activity to a propionate-infused colon was extremely variable, which indicates that these two molecules act through separate pathways on enteroendocrine cells, and not necessarily through GPR receptors. Another target for butyrate is the Olf558 receptor, which has been colocalized to 5-HT-containing enteroendocrine cells (Bellono et al., 2017; Vincent et al., 2018). Since butyrate exists in equivalent concentrations and acts with a similar affinity toward this receptor, it may serve as a competitive antagonist to propionate on GPR43s. A decrease in PYY due to altered G protein coupling could be enough to lessen propionate’s inhibitory effects, even if 5-HT was not directly released by the binding of butyrate. Thus, the complicated interactions between these two SCFAs may be explained by (a) competitiveness for GPR receptors and (b) two different activating receptors on enteroendocrine cells, which ultimately release separate signaling molecules to act on sensory neurons in the myenteric plexus. Overall, the strongest inducer of CMCs in all conditions was mechanical distention.

### Butyrate Augments Two Types of Motor Activity Associated With Pellet Propulsion

In the present study, pellets were propelled distally by two distinct mechanisms. One was the CMC; although not initiated by pellets, a CMC was able to move a pellet distally and 2–5 CMCs could expel the pellet from the colon. This intermittent propulsion by CMCs was also observed in the guinea pig colon (Costa et al., 2019). Weak CMCs increased in amplitude upon reaching the portion of the colon that was distended by the pellet (Figure 14). Pellets usually moved slightly orally caused by the relaxation phase of the CMCs. Similar to its effects with general distention, 10 mM butyrate had an excitatory but inconsistent effect on the frequency of CMCs.

Another distinct motor pattern appeared only after pellet insertion, which was the peristaltic reflex initated by a pellet. A reflex often started with circular muscle contraction just oral to the pellet which then moved downward over a prolonged period of time. Unlike CMCs, they were caused directly by localized distention and are explained by the “law of the intestine”: the peristaltic reflex seems to function via Bayliss and Starling’s principles where the polarization of intrinsic neural pathways results in oral contraction and anal relaxation upon enteric mechano-sensory neural excitation (Bayliss and Starling, 1900; Costa and Furness, 1976). This motor pattern is a reflex in that it is initiated by pellet-induced stretching but is a complex process influenced by bolus size and pellet consistency (Costa et al., 2017). Furthermore, the reflex is influenced by luminal factors: the present study shows that both Krebs perfusion and 10 mM butyrate greatly augmented the peristaltic reflexes generated.

Overall, localized distention did not seem to diminish butyrate’s stimulatory capacity to the same degree as general distention. Butyrate augmented both CMCs and peristaltic reflexes to decrease the amount of time required to expel each pellet. Additionally, fewer movements were required to expel each pellet, indicating better coordination of motility patterns to increase the effectiveness of peristalsis.

## Data Availability Statement

The datasets generated for this study are available on request to the corresponding author.

## Ethics Statement

The animal study was reviewed and approved by the McMaster University Animal Research Ethics Board.

## Author Contributions

JH and J-HC designed the study and supervised execution and analysis. WT did most of the experimentation and analysis and wrote the first draft of manuscript. GL conducted the propionate experiments and made a substantial contribution to analysis and manuscript writing. All authors approved the final version.

## Conflict of Interest

The authors declare that the research was conducted in the absence of any commercial or financial relationships that could be construed as a potential conflict of interest.
